# Wastewater Management under Global Climate Change Conditions

**DOI:** 10.1021/acsenvironau.6c00097

**Published:** 2026-05-18

**Authors:** Tao Liu, Glen T. Daigger, Xiangdong Li

**Affiliations:** † State Key Laboratory of Climate Resilience for Coastal Cities, Department of Civil and Environmental Engineering, 26680 The Hong Kong Polytechnic University, Hung Hom, Kowloon, Hong Kong SAR, China; ‡ Research Institute for Sustainable Urban Development (RISUD), The Hong Kong Polytechnic University, Hung Hom, Kowloon , Hong Kong SAR, China; § Department of Civil and Environmental Engineering, 122391 University of Michigan, Ann Arbor, Michigan MI 48109, United States

**Keywords:** climate change, wastewater management, greenhouse gas emissions, resource recovery, decentralized treatment, resilience

## Abstract

Urban wastewater systems were historically designed under assumptions of climatic stationarity; however, climate change is rapidly altering the boundary conditions under which they operate. In this Perspective, we synthesize emerging evidence to conceptualize wastewater systems as climate-responsive infrastructure embedded within dynamic environmental feedbacks. We propose an input–output boundary framework to characterize climate change-driven alterations in wastewater systems. On the input side, climate change affects wastewater systems through three primary pathways: increased flow variability, shifts in physicochemical characteristics of wastewater, and changes in the contaminant spectrum. On the output side, increased risks of untreated discharge or improperly treated effluents and amplified greenhouse gas emissions intensify the environmental footprint of wastewater systems. Collectively, these nonstationary pressures undermine the reliability and effectiveness of key wastewater components, such as wastewater collection networks, biological processes, and advanced treatment units, thereby challenging conventional design and operation. Finally, we highlight four key opportunities of climate-resilient wastewater management, including predictive modeling and adaptive operational strategies enabled by digitalization, resilient infrastructure and process design, decentralized treatment with resource recovery, and nature-based solutions. Reframing wastewater systems as adaptive, low-emission, and circular infrastructures is essential to sustain urban water security under accelerating climate change.

## Graphical abstract

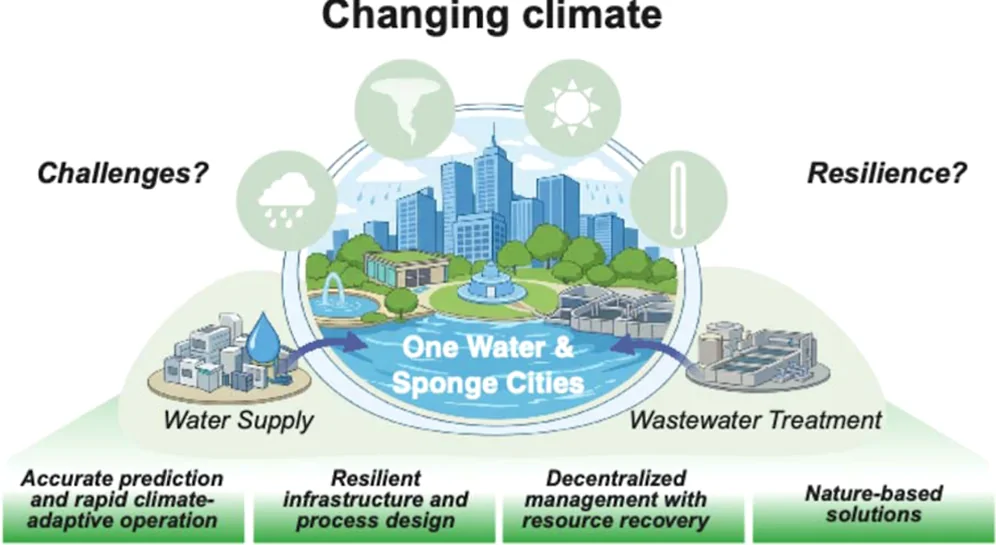

## 1 Climate Change-Driven Alterations to the Boundary Conditions of Wastewater Systems

Rapid global socioeconomic development over the past century has coincided with a pronounced global warming trend. Assessments by the Intergovernmental Panel on Climate Change (IPCC) consistently document rising global mean temperatures, accelerating loss of snow and ice cover, and increasing instability in atmospheric and oceanic circulation patterns.^1^ The frequency and intensity of extreme weather events, including heatwaves, intense rainfall, droughts, and tropical cyclones, have risen markedly, with cascading impacts on ecosystems, food security, urban infrastructure, and public health. The 2023 IPCC assessment projects that global mean sea-level rise will approach 0.5 m by midcentury, potentially exposing more than 800 million people in over 500 coastal cities to recurrent flooding and extreme weather, with annual global economic losses exceeding US$1 trillion.^2^ In response, governments worldwide are advancing national adaptation strategies. Examples include China’s National Climate Change Adaptation Strategy 2035,^3^ the European Union Adaptation Strategy,^4^ and the United States National Climate Adaptation and Resilience Strategy.^5^ These initiatives collectively highlight the urgency of strengthening critical urban infrastructures under a rapidly changing climate.

Urban wastewater systems, a critical component of urban infrastructure, were historically designed under major assumptions of climatic stationarity. However, climate change now alters both the input and output boundary conditions under which these systems operate (Figure 1). On the input side, hydrological variability is intensifying, with more frequent extreme rainfall events that generate hydraulic surges and overflow risks and accelerating sea-level rise that exacerbates tidal intrusion and groundwater seepage into sewer networks. Second, the physicochemical characteristics of wastewater have been fundamentally altered. Salinity intrusion alters ionic strength and buffering capacity, elevated temperatures accelerate biochemical reaction kinetics and microbial turnover, and more frequent redox variations may disturb established functional microbial communities. Third, the spectrum of environmental contaminants in wastewater is likewise evolving.^6^ Extreme events mobilize legacy pollutants when hydrological thresholds are exceeded; drought-induced dilution loss elevates contaminant concentrations; rising temperatures stimulate pathogen proliferation; and altered environmental conditions may promote chemical and biochemical transformations that generate new or more toxic compounds.

**Figure 1:**
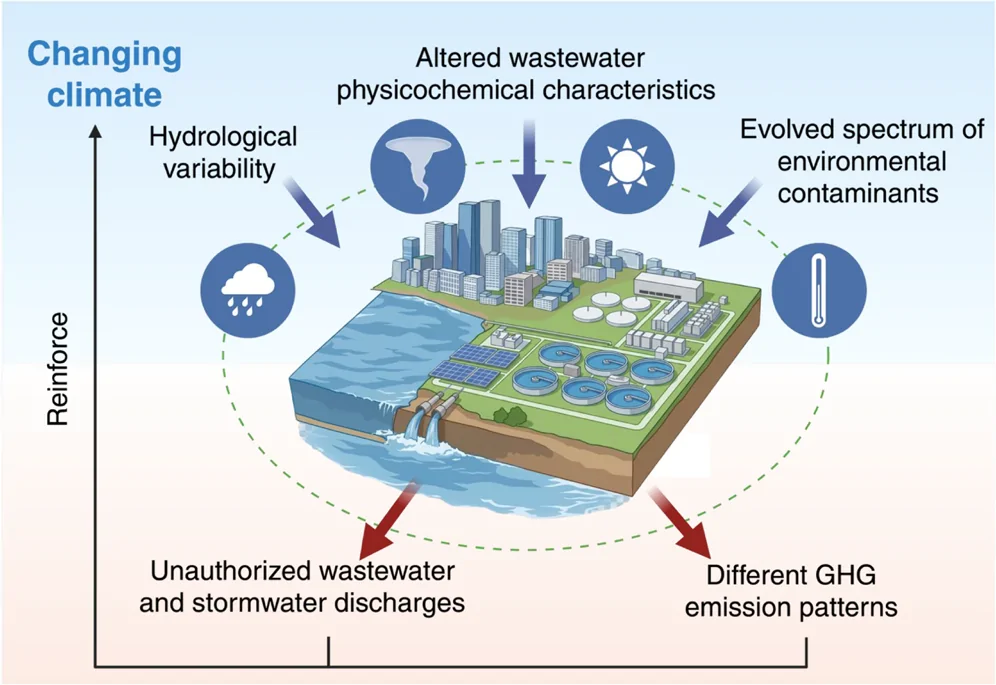
Climate change-driven nonstationary boundary conditions of wastewater systems.

In parallel, output boundary conditions of wastewater systems are also affected (Figure 1). Nonstationary climatic events increase the likelihood of untreated or inadequately treated wastewater discharges. For instance, climate change-amplified atmospheric river events in the United States in late 2022 resulted in more than 227,000 m^3^ of unauthorized wastewater and stormwater discharges, which introduced pathogens, nutrients, and biodegradable organics into receiving waters.^7^ Meanwhile, rising temperatures and wastewater salinities reduce the solubility of greenhouse gases (GHG), such as methane and nitrous oxide, shifting the gas–liquid equilibrium toward the gaseous phase and increasing emissions. These altered output boundary conditions of wastewater systems can, in turn, reinforce climate forcing, thereby creating a self-amplifying feedback loop between wastewater systems and climate change.

## 2 Challenges of Urban Wastewater Systems under Global Climate Change

Intensified hydrological extremes, sea-level rise, elevated temperatures, and shifting contaminant profiles collectively introduce nonstationary, highly dynamic operating conditions for urban wastewater systems. Within the input–output boundary framework, these pressures reshape both the input conditions of wastewater systems by altering influent quantity, composition, salinity, temperature, and contaminant profiles and the output conditions by changing the discharge risks, GHG emissions, and performance expectations that determine acceptable effluent quality and system emissions. These changes alter both the quantity and quality of influent streams while simultaneously affecting treatment kinetics, infrastructure durability, and compliance with effluent discharge requirements. Exposure to such nonstationary boundary conditions fundamentally challenges the stability, resilience, and reliability of key components across urban wastewater collection and treatment systems.

- Under nonstationary boundary conditions, wastewater collection systems experience intensified hydraulic and chemical stresses.^8^ Under extreme weather conditions, inflow and infiltration (I/I) can exceed average wastewater flow by an order of magnitude,^9,10^ exceeding the design capacity and leading to sewer overflows, environmental pollution, public health risks, infrastructure damage, and downstream overloading of wastewater treatment plants.^11^ Moreover, increased hydraulic flow can resuspend and transport previously deposited solids in sewer systems, thereby generating a transient surge in influent solids loading to wastewater treatment plants, known as the “first flush” effect.^12^ Concurrently, concrete corrosion may be exacerbated by tidal intrusion and groundwater seepage into sewer networks, shortening infrastructure lifespan and compromising structural integrity. In sewers, concrete deterioration is primarily caused by sulfuric acid produced by sulfur-cycling microorganisms.^13^ Thus, the elevated sulfate levels in wastewater resulting from salinity intrusion will, in theory, accelerate corrosion. In addition, chloride intrusion promotes reinforcement corrosion by penetrating the concrete matrix, breaking down the passive layer on embedded steel, and accelerating electrochemical reactions, thereby acting synergistically with sulfate-driven biogenic corrosion to exacerbate structural degradation.^14^
- Biological processes face reduced functional robustness and increased operational uncertainty under nonstationary boundary conditions. Extreme rainfall events introduce shock hydraulic loading and sudden dilution, disrupting sludge retention, and increasing the risk of biomass washout.^15–17^ As a result, secondary clarifiers become critical failure points, where hydraulic surges compromise sludge settleability and effluent quality. Salinity intrusion and temperature elevation can also affect the microbial community structure of biological processes. For example, sudden increases or decreases in salinity, caused by seawater intrusion and extreme weather events, can destabilize biological performance and require months to recover. Moreover, salinity may also affect biogenic GHG emissions by (1) shifting microbial communities (e.g., elevated sulfate may promote sulfate-reducing bacteria over methanogens, reducing methane production in sewers),^18^ (2) physicochemical controls on gas transfer (e.g., methane solubility decreases by ∼5% as salinity increases from 0% to 1%,^19^ facilitating its emissions from the liquid to gas phase), and (3) microbial pathway regulation (e.g., salinity may disrupt nitrification/denitrification, leading to nitrous oxide accumulation).^20^ For instance, a recent study estimated total methane production from global sewer networks at 1.18–1.95 Tg CH_4_ yr^–1^.^21^ Assuming that methane production and other transport conditions remain unchanged, a salinity increase from 0% to 1% could reduce methane solubility and thereby enhance gas–liquid partitioning, potentially increasing methane emissions by 0.012–0.049 Tg CH_4_ yr^–1^. Collectively, these pressures undermine the stability, efficiency, and predictability of biological processes.
- Tertiary treatment also faces operational challenges. Extreme rainfall events introduce hydraulic fluctuations and suspended solids carryover, imposing substantial stress on downstream tertiary processes. Increased particulate loading accelerates membrane fouling and filter clogging,^22^ reduces UV transmittance and pathogen inactivation efficiency due to particle shielding,^23^ elevates disinfectant demand,^24^ and enhances oxidant scavenging in advanced oxidation processes.^25^ Elevated temperatures and altered ionic strength and buffering capacity of wastewater may also alter reaction kinetics in advanced oxidation processes and affect disinfectant decay rates.^26^ Furthermore, more emerging contaminants may be present in wastewater due to climate-driven mobilization of legacy pollutants,^27^ increased road runoff from extreme rainfall events,^28^ and intensified urban chemical use associated with climate adaptation measures (e.g., fire retardants may enter stormwater systems after rainfall events).^29^ These cascading effects may increase the operational energy demand, chemical consumption, and performance variability of tertiary treatment under climate uncertainty.

## 3 Climate-Resilient Wastewater Management

### 3.1 Accurate Prediction and Rapid Climate-Adaptive Operation

In the era of digitalization, climate-resilient wastewater management can be supported by predictive modeling and adaptive operational strategies, particularly in response to increasingly frequent changes in the input boundary conditions of wastewater systems (Figure 2). The integration of climate forecasting, real-time monitoring, and machine learning approaches enables continuous system diagnosis, rapid anomaly detection, performance forecasting, asset condition assessment, and optimized operation and maintenance. For instance, the strong predictive capability of long short-term memory (LSTM) in sewer flow modeling has been applied to optimize in-sewer storage control for overflow mitigation and downstream plant load balancing under extreme weather conditions.^30,31^ Recurrent neural networks (RNN)–restricted Boltzmann machine (RBM) frameworks have been developed to predict multivariate influent water-quality time series and facilitate real-time anomaly detection, enabling early identification of abnormal influent conditions that may compromise operational resilience.^32^ Hybrid convolutional neural networks (CNN)–LSTM models trained on high-frequency operational data sets have achieved accurate organic mass-flow prediction, supporting feedforward aeration and chemical-dosing control strategies that enhance the system resilience.^33^ More recently, applications of deep reinforcement learning (DRL) in flood mitigation and combined sewer overflow (CSO) reduction have demonstrated improved performance and a faster control response under variable climatic scenarios.^34,35^ Collectively, supported by large volumes of high-quality data and model-enabled soft sensing and predictive analytics,^36,37^ these tools enhance early warning capability, operational flexibility, and disturbance recovery for the urban wastewater systems and support proactive operational decision-making. These capabilities can be further augmented by large language model (LLM)-based agents for automated analysis and control assistance.^38^

**Figure 2:**
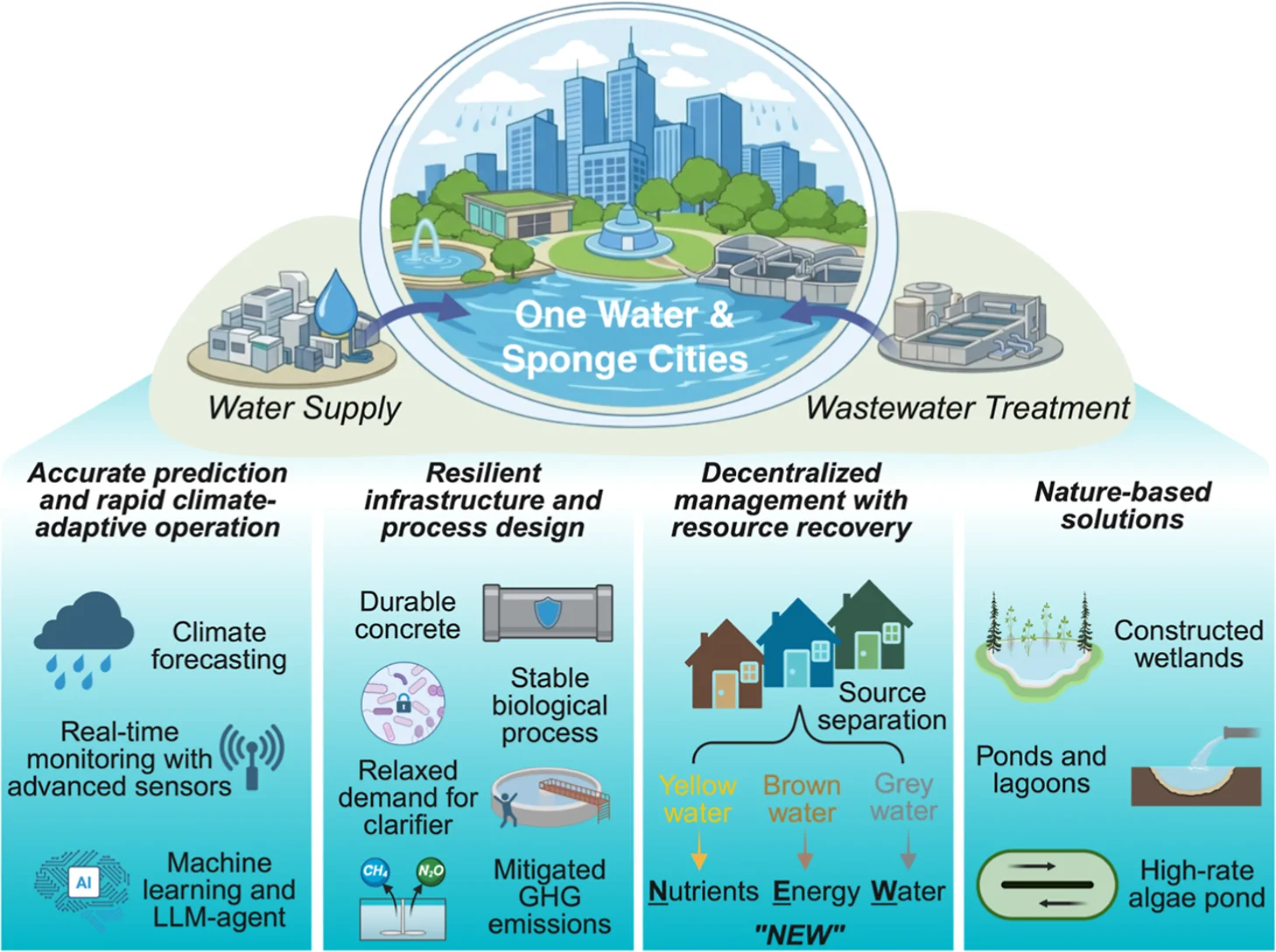
Climate-resilient wastewater management.

### 3.2 Resilient Infrastructure and Process Design

Design-wise resilience emphasizes durable infrastructure materials and robust biological processes capable of withstanding altered input boundary conditions of wastewater systems (Figure 2), including hydraulic shocks, salinity intrusion, and temperature variability. For infrastructure materials, the development of innovative low-carbon concretes with enhanced resistance to microbiologically induced concrete corrosion (MICC) and sustained antimicrobial functionality is necessary. Compared with conventional ordinary Portland cement, low-calcium binders, such as slag- and fly ash-based alkali-activated materials and geopolymer concretes, exhibit superior stability under aggressive acidic, high-chloride, and high-sulfate conditions.^39^ Their primary reaction products, including sodium/calcium aluminosilicate hydrates and layered double hydroxides (LDHs), provide a chemically resilient matrix with a lower calcium hydroxide content and reduced susceptibility to acid dissolution.^40^ Additionally, nitrite-based or other protective agents can be loaded into aggregate pore networks, enabling controlled release under corrosive conditions.^41^ Furthermore, biomineralization strategies, such as microbially induced carbonate precipitation, offer a complementary approach to enhancing concrete densification, crack healing, and long-term durability.^42^

In biological processes, as elaborated above, one of the most critical failure points under extreme climatic events is the secondary clarifier, which frequently loses its solid–liquid separation capacity. Accordingly, treatment configurations that decouple solid retention time (SRT) from hydraulic retention time (HRT) offer enhanced functional robustness. Biofilm- and granule-based systems, such as moving bed biofilm reactors (MBBRs),^43^ membrane-aerated biofilm reactors (MABRs),^44^ and aerobic granular sludge (AGS),^45^ maintain effective SRTs relatively independent of hydraulic fluctuations, conferring resistance to biomass washout under shock-loading conditions and more stable performance during extreme rainfall events. For example, MBBRs maintained resilient ammonia and organic removal under various representative wet-weather disturbance scenarios:^43^ (1) high hydraulic flow and elevated organic loading (simulating flooding and first-flush conditions); (2) high flow and loading combined with dissolved oxygen depletion (simulating flooding accompanied by power outage); and (3) starvation under anoxic conditions (simulating temporary plant shutdown). Beyond hydraulic perturbations, microbial resilience to salinity fluctuations is also critical. Biofilm systems and granular sludge processes often exhibit enhanced tolerance to salinity shifts due to the protective extracellular polymeric substance (EPS) matrix.^46,47^ Furthermore, recovery of treatment performance following incidental biomass loss and activity suppression should proceed in a staged, well-controlled manner. The immediate priority is to stabilize the system and prevent further biomass loss by increasing sludge return rates and, where possible, utilizing equalization capacity to control wastewater flow and influent salinity. Operational parameters, such as dissolved oxygen, could be leveraged to create a favorable environment for microbial regrowth.^48^

### 3.3 Decentralized Management with Resource Recovery

Decentralized treatment and satellite facilities improve operational flexibility, shorten conveyance distances, and mitigate overflow risks during storm events (Figure 2).^49–51^ By redistributing treatment closer to wastewater generation points, these systems enhance the resilience of wastewater systems to the climate change-altered input boundary conditions, such as hydrological surges and contaminant fluctuations, while also lowering the risk of adverse output boundary outcomes, including untreated overflows and compromised effluent compliance. Modular designs further enable phased capacity expansion and faster recovery following climate-induced disruptions.^52,53^ Decentralized wastewater treatment can operate as nongrid, small-grid, and hybrid systems that complement centralized infrastructure,^51^ with pilot demonstrations achieved in China,^54^ the US,^55^ Europe,^56,57^ and Australia.^58^ Moreover, decentralized systems create favorable conditions for resource recovery, including nutrients, energy, and water (as the NEW concept), further enhancing the system-level resilience. In centralized systems, the common practice of combining all liquid waste streams, including urine, fecal wastewater, greywater, and, in many cases, stormwater in combined sewer systems, results in strong dilution of nutrients and energy carriers, thereby making energy and nutrient recovery technically challenging and economically inefficient.^59^ In contrast, decentralized systems enable targeted collection and treatment of specific wastewater fractions, greatly improving the feasibility and efficiency of resource recovery. For example, Garrido-Baserba et al. modeled a distributed treatment system for a representative 2000-person urban block that integrates urine diversion, anaerobic digestion of blackwater with struvite recovery, and membrane-based treatment of greywater.^60^ By diverting and treating waste streams locally, such systems reduce flows to centralized sewer networks while enhancing resource recovery, indicating approximately 2-fold higher energy and nitrogen recovery compared with conventional centralized treatment.

Beyond energy and nutrient recovery, decentralized and hybrid systems are increasingly critical in the context of declining reliability of conventional water supplies under climate change. More frequent and prolonged droughts, together with the growing intensity and intermittency of rainfall events, limit both water storage capacity and the effective capture of precipitation.^61^ Therefore, alternative water sources, particularly water reuse and desalination, are becoming essential. Notably, from a process and economic perspective, water reuse may be more favorable than desalination. As both reuse and desalination rely heavily on membrane processes, the lower total dissolved solids (TDS) of reuse streams enables substantially higher water recovery efficiencies (typically ∼85% for reuse versus ∼55% for desalination), resulting in lower energy demand and operational costs. Recent modeling studies suggest that hybrid urban water systems integrating centralized infrastructure with distributed reuse nodes can exhibit lower disruption severity, smaller impact ranges, and faster recovery following infrastructure failures.^62^ By diversifying supply nodes and reducing reliance on single-point infrastructure, such configurations enhance the capacity of urban water systems to absorb and recover from disturbances.

Furthermore, some unique resource recovery opportunities in saline wastewater should be given attention. Wastewater salinization is becoming increasingly common in coastal cities under climate change through both planned (e.g., intentional use of seawater for toilet flushing) and unplanned (e.g., seawater intrusion, tidal backflow, and storm-induced hydraulic disturbances) pathways. For example, in Hong Kong, due to the lack of freshwater, toilet flushing with seawater results in sulfate concentrations above 200 mg S/L and chloride concentrations above 5000 mg Cl/L, creating emerging opportunities for their recovery. For instance, sulfate can be converted to elemental sulfur and sulfide through biological or electrochemical processes, enabling its potential in situ use as an electron donor for denitrification,^63^ while high chloride concentrations enable electrochemical chlorine and chloramine generation for on-site disinfection and membrane fouling control.^64^ In addition, multivalent ions such as calcium and magnesium promote mineral precipitation, facilitating nutrient recovery and the formation of value-added mineral products. Another emerging opportunity is the recovery of so-called “blue energy”, which exploits the chemical potential difference between water streams with different salinities.^65^ Despite the potential of this new source of clean, renewable energy, its large-scale exploitation remains limited due to the low efficiency of current conversion schemes.

### 3.4 Nature-Based Solutions

Nature-based solutions (NBS) provide an important pathway to enhance the resilience of wastewater treatment systems by employing ecosystem processes for pollutant removal while reducing dependence on energy-intensive infrastructure (Figure 2).^66^ By buffering climate change-altered input boundary conditions, such as hydraulic surges and temperature variability, NBS can also moderate adverse output boundary conditions by reducing overflow risks, stabilizing effluent quality, and expanding opportunities for water reuse. These systems, including constructed wetlands,^67^ soil infiltration systems,^68^ wastewater stabilization ponds,^69^ and high-rate algal ponds,^70^ mimic natural biogeochemical cycles, in which interactions among vegetation, porous media, and diverse microbial communities jointly drive wastewater purification. A key resilience advantage of NBS lies in their operational simplicity and ecological redundancy.^66^ Compared with conventional mechanized treatment processes, NBS rely less on complex mechanical equipment and continuous energy inputs,^71^ making them less vulnerable to power outages, equipment failures, or operational disruptions. The ecological complexity within these systems, including stratified microbial communities and plant–microbe interactions, also provides functional redundancy, enabling treatment processes to persist under fluctuating hydraulic and environmental conditions. Beyond treatment reliability, NBS contribute to broader urban water system resilience by integrating wastewater management with ecosystem functions. Vegetated systems, such as constructed wetlands, can attenuate stormwater peaks through infiltration, retention, and delayed runoff, thereby buffering hydraulic shocks to the sewer and treatment infrastructure.^72^ At the same time, they provide additional ecosystem services, such as carbon sequestration, biodiversity support, social services, and opportunities for water reuse.^73,74^ By embedding treatment processes within multifunctional landscapes, NBS transform wastewater infrastructure from single-purpose facilities into adaptive systems capable of responding to climatic, hydraulic, and environmental disturbances.^75^

## 4 Water and Wastewater Research in ACS Environmental Au

Since its launch in 2021, *ACS Environmental Au* has aimed to advance environmental science and engineering through high-quality research spanning a wide range of environmental topics. Within this broad scope, water and wastewater research is a recurring theme in the journal. While we are very interested in research areas such as membrane-based treatment^76,77^ and the fate, risk, and removal of emerging contaminants in water and wastewater systems,^78,79^ recent contributions in *ACS Environmental Au* further reflect research directions closely aligned with climate-resilient wastewater management. For accurate prediction and rapid climate-adaptive operation, machine learning-based soft sensors for onsite wastewater treatment systems demonstrated the potential of data-driven tools to predict water quality and support adaptive operation under dynamic conditions.^36^ Additionally, several studies highlighted the role of decentralized management with resource recovery in improving treatment robustness and sustainability under variable environmental conditions. For instance, multicriteria decision frameworks provided approaches to evaluate the contextualized sustainability of sanitation and resource recovery technologies;^80^ analyses of nonsewered sanitation systems^81^ and fecal sludge treatment with pyrolysis^82^ highlighted the importance of decentralized treatment solutions for improving resilience and circular resource management; and an electrochemical stripping system was demonstrated as a potential technology for recovering ammonia from source-separated urine, achieving high nitrogen removal and recovery efficiencies.^83^ Nature-based solutions for wastewater treatment are also an important research direction of the journal. For example, vertical-flow constructed wetlands amended with Fe­(III)-EDTA demonstrated enhanced removal of wastewater-derived trace organic contaminants;^84^ long-term analyses of microbial community dynamics in constructed wetlands revealed how spatial and temporal microbial shifts support treatment stability under fluctuating conditions;^85^ and a perspective paper argued that constructed wetlands could offer a low-cost, publicly acceptable, multibeneficial solution for treating reverse osmosis concentrate in expanding wastewater reuse systems.^86^ Collectively, these studies reinforce the broader perspective outlined in this review: addressing the challenges posed by climate change requires moving beyond conventional wastewater treatment toward integrated, adaptive, and resource-oriented water systems. By combining advances in materials science, microbial ecology, digital monitoring, decentralized sanitation technologies, and sustainability assessment, recent research demonstrates how wastewater systems can evolve from linear pollution-control infrastructures into resilient, circular, and climate-responsive urban water systems.

Nevertheless, the implementation of the approaches to enhance the resilience of urban wastewater systems must be evaluated not only by their technical promise but also by their cost, scalability, and context-specific feasibility. Accurate prediction and rapid climate-adaptive operation may offer relatively readily implementable strategies by improving the use of existing infrastructure, but their effectiveness depends on sensor reliability, data availability, model transferability, and institutional capacity for real-time decision-making. A resilient infrastructure and process design can provide stronger protection against hydrological and physicochemical shocks, yet full-scale retrofits or system-wide redesigns are capital-intensive and must be justified by clear gains in risk reduction, treatment stability, and asset longevity. Decentralized management with resource recovery may reduce conveyance burdens and improve local circularity, but its deployment requires careful consideration of maintenance responsibility, product-market development, regulatory acceptance, and economies of scale. Similarly, nature-based solutions can enhance buffering capacity and deliver cobenefits, such as flood mitigation and ecological restoration, but their performance is often constrained by space requirements, seasonal variability, and maintenance needs. Therefore, future research and demonstration projects should move beyond demonstrating that emerging technologies are “better” in principle and instead quantify under what conditions they deliver meaningful resilience benefits relative to their life-cycle costs, implementation complexity, and scalability across diverse urban wastewater contexts.

Climate change is redefining urban wastewater systems by introducing nonstationary boundary conditions that simultaneously alter influent characteristics and elevate downstream effluent risks. Hydrological extremes, altered physicochemical characteristics, and the evolving contaminant spectra of wastewater increasingly challenge collection networks, biological treatment, polishing processes, and overall operational stability, exposing the limitations of infrastructure designed under the assumption of climatic stationarity. In this context, the imperative to respond to climate change should not be viewed solely as a burden but also as an opportunity and a driver to move beyond the century-old approaches toward newer, climate-responsive, and resilient approaches. Such a transition is essential not only for protecting environmental and public health but also for advancing the Sustainable Development Goals, particularly SDG 6 (Clean Water and Sanitation), SDG 11 (Sustainable Cities and Communities), SDG 12 (Responsible Consumption and Production), and SDG 13 (Climate Action). Beyond improvements in individual technologies, systems-level thinking is critical. For instance, the One Water paradigm promotes coordinated management of drinking water, wastewater, stormwater, and reuse as parts of a single interconnected system; and the Sponge Cities program emphasizes nature-based, hydrologically restorative infrastructure for stormwater retention, infiltration, and flood adaptation. Together, these frameworks point toward integrated, adaptive, and nature-based urban water systems capable of delivering long-term water security, climate resilience, and environmental sustainability.
